# Vital stress in animals and plants

**DOI:** 10.1007/s00709-024-02001-5

**Published:** 2024-10-17

**Authors:** Peter Nick

**Affiliations:** Joseph Gottlieb Kölreuter Institute for Plant Sciences, Karlsruhe Institute of Technology, Karlsruhe, Germany

Only rarely are the conditions of the outer world in harmony with our inner requirements. The tension between environment and internal homeostasis is known as stress, and the ability to cope with this dissonance has decided the survival of life on our planet from its very beginning. This is even more valid if one considers that homeostasis, by its very nature, is a cybernetic process and not a static condition. Even under “normal” conditions the changes inflicted by ongoing development mean that the actual situation deviates from equilibrium. The ability to efficiently sense such deviations and deploy adaptive responses culminating in a new homeostasis has been a central strategy for stress resilience. Thus, even under healthy conditions, organisms continuously oscillate around their ideal state, they never rest. These small deviations are not only tolerated; they even are required for health, a phenomenon termed as eustress (Selye 1973). Only, when the challenges exhaust the adaptive abilities of the organism, such that it fails to establish a new dynamic equilibrium, stress becomes destructive (distress *in senso* Seyle, 1973). The regulation of cell division, probably the most evident manifestation of life activity, can serve as a paradigm illustrating the importance of eustress. In many eukaryotic organisms, the progression through the cell cycle is linked to specific stress responses, not negatively, but positively. Two contributions to the current issue, one from animals, and one from plants, address different facets of this vital stress form.

The contribution by Wu et al. (2024) deals with the role of the autophagy-related protein ATG8 in the microtubular organisation of ciliates. They use the large-celled *Euplotes* as an experimental model, making use of the knowledge on tubulins gained in other ciliates, like *Tetrahymena* and *Paramecium* (for review see Libusová and Draber, 2006). In animal cells, microtubules are usually organised on centrioles, and the basal bodies of cilia and flagella have derived from centrioles leading to the question, how nucleation of mitotic microtubule structures and microtubule arrays with a role in interphase (cilia and flagella) are differentiated. Here, the protein Oral-Facial-Digital Syndrome Protein 1 is crucial, since this protein can silence the nucleation of microtubules from centrioles or basal bodies. By selective proteolytic decay of this protein at the basal body, the block is released and cilia form. This decay is mediated by ATG8, a protein central to the formation of autophagosome membranes in response to stress. This ubiquitin-like protein can recruit its target to degradation in the proteasome. By immunolabelling, the authors can show that ATG8 is localised to the basal body of *Euplotes*, their model system. When they intercept the expression of *ATG8* with a specific miRNA, this localisation is lost and the downstream target cilia transport protein IFT88 is degraded proteolytically, culminating in perturbed ciliogenesis and reduced swimming activity. Since the organisation of cilia is accompanied by differential post-translational modifications of the constituting tubulins (Libusová et al. 2005), ATG8 might interfere with the activity of the corresponding tubulin-modifying enzymes. The work shows, how a protein that originally is functioning in autophagy, a stress-related phenomenon, can be recruited to confer a specific breakdown of negative regulators and, thus, to uncouple tubulin nucleation from its otherwise dominating role in mitosis.

Also, the contribution by Hernández-Esquivel et al. (2024) shows that stress signals can play a positive role for development, this time in plants. Induction of lateral roots is central for the formation of a vigorous root system and initiates with the commitment of silent cells in the pericycle layer for initiation of a stem cell niche and the generation of a meristem that will drive the growth of lateral roots. This initiation event comes with an activation of mitogen-activated kinase cascades that respond to a local oxidative burst provided by the plasma-membrane located NADPH oxidase respiratory burst oxidase homologue, a central input for plant-stress signalling. How the formation of reactive oxygen species at the plasma membrane is transduced into a phosphorylation cascade transmitting the signal into the nucleus, where gene activation is deployed, remains poorly understood. In mammalian cells, target of rapamycin (TOR), a member of the phosphoinositide 3-kinase-related kinase family, has been shown to be central (reviewed in Wullschleger et al. 2006). The activity of this pathway is regulated by the redox status of the cell, whereby reducing conditions inhibit, and oxidative conditions activate. Some components of the kinase complex are conserved in plants, and the authors ask whether a similar mechanism is at work in lateral root formation. In fact, they can induce lateral root formation by exogenous hydrogen peroxide in the model plant *Arabidopsis thaliana*, and this correlates with the expression of cyclins as well as of the plant TOR homologue (probably as a secondary feedback response to the activation of this pathway). When they scavenge endogenous reactive oxygen species by ascorbic acid, they block TOR expression as well as lateral root formation. Likewise, inihibition of TOR activity by torin, not only inhibits expression of a ribosomal protein that otherwise is induced by the TOR pathway. Torin also blocks the stimulation of lateral root formation by hydrogen peroxide. Thus, activation of TOR is a necessary event for the induction of lateral roots in response to oxidative burst.

Whether stress promotes the formation of a stem-cell niche depends on the functional context, even in the same organism. This is illustrated by the contribution of Song et al. (2024). Here, the effect of the phytohormone abscisic acid on the formation of a shoot stem-cell niche is considered. Abscisic acid is a central player in the stress response of plants and basically slows down metabolism, minimizing the risk of generating reactive oxygen species from perturbed electron transport across the inner membranes of mitochondria and plastids. Interestingly, its synthesis is deployed by reactive oxygen species (Xiong and Zhu 2003). Plants are principally endowed with totipotency, meaning that by hormonal treatment it is possible to generate embryos from somatic cells. In this context, the ratio of auxins (favouring root development) and cytokinins (favouring shoot development) is crucial. The formation of the shoot stem-cell niche is under the control of the transcription factor *WUSCHEL*. This master switch is, in turn, regulated by cytokinin signalling leading to specific histone modifications. Exogenous ABA can block shoot regeneration, and the authors use a combination of chromatin immunoprecipitation, gene expression analysis, and promoter-reporter localisation studies to show that ABA inhibits both, the histone modifications, and the induction of *WUSCHEL* disrupting the formation of a stem-cell niche. The contrast with lateral root formation may appear difficult to understand. However, one needs to know that stress in land plants is linked to limited water availability in most cases. Here, the plant needs to render vital decisions. In the aerial organs, perturbations of photosynthesis will lead to a considerable oxidative imbalance that can affect the entire plant and will bind considerable resources for being mitigated, for instance by the synthesis of enzymatic and non-enzymatic antioxidants. To shift resources into the formation of additional lateral roots represents a meaningful strategy because it will allow to recruit additional supply of water.

Although the biological context of these three examples is different, there is a common theme: individual aspects in the cellular consequences of stress are used as signals to deploy cellular change that can be either part of normal development (as in case of the ciliogenesis) or even of adaptive nature (as in case of the plant stem-cell niche). In *Euplotes*, a component of the stress-induced autophagosome is recruited to release microtubule nucleation specifically at the basal body while the centrosome remains silent. Lateral roots as important factor for plant resilience to water scarcity are deployed by an oxidative burst that activates the formation of a stem-cell niche through the TOR pathway. In contrast, the formation of a stem-cell niche in the shoot is suppressed by abscisic acid by specifically targeting a histone modification that otherwise would allow the induction of WUSCHEL as a master switch for stem-cell formation and maintenance. Again, there is a link with oxidative stress, however, inversely to the lateral root, because reactive oxygen species deploy abscisic acid as inhibitor (contrasting with TOR as activator) of stem-cell fate. This inversion is linked with adaptive stress strategies of land plants because partitioning of resources from the shoot to the root represents an efficient strategy to cope with water shortages. All three cases show paradigmatically that stress (if integrated into and confined by a functional context) can be vital.
